# A randomized, double-blind, placebo-controlled study to evaluate the benefits of a standardized *Nigella sativa* oil containing 5% thymoquinone in reducing the symptoms of seasonal allergy

**DOI:** 10.1097/MD.0000000000039243

**Published:** 2024-08-09

**Authors:** Anju Majeed, Shaheen Majeed, Avinash Kadasiddappa Parameswarappa, Avinash Murali, Satish Gudimallam, Chikkalingaiah Siddegowda, Harshith Chandrashekar, Lakshmi Mundkur

**Affiliations:** a Sami-Sabinsa Group Limited, Bangalore, Karnataka, India; b Sabinsa Corporation, East Windsor, NJ; c Medstar Speciality Hospital, Bangalore, Karnataka, India; d BGS Global Institute of Medical Science Sunkalpalya, Bengaluru, Karnataka, India.

**Keywords:** allergic rhinitis, Immunoglobulin E, Patient Global Impression of Change, randomized controlled trial, thymoquinone, total nasal symptom score, total ocular symptom scale

## Abstract

**Background:**

Allergic rhinitis (AR) or seasonal allergy characterized by sneezing, nasal congestion, nasal itching, and nasal discharge, triggered by immune reactions to environmental allergens. Present day customers also monitor the personal improvements in the area of Evidence-Based natural medicines/supplements

**Methods:**

A randomized, double-blind, placebo-controlled study was conducted on 65 participants aged 18 to 60 years having 2 or more allergic symptoms like sneezing, rhinorrhoea, nasal obstruction, and nasal itching for a cumulative period greater than 1 hour per day. The study participants received a capsule of NSO (250 mg) with 2.5 mg piperine (BioPerine) as a bioavailability enhancer or a placebo, twice a day, after food for 15 days. The primary objectives were evaluated by mean change in Total Nasal Symptom Score and the duration of AR symptoms per day from baseline to Day 15. Secondary endpoints were changes in Total Ocular Symptoms Score, AR symptom frequency and severity, serum Immunoglobulin E levels, and Patient Global Impression of Change scale. Adverse events were monitored throughout the study.

**Results:**

Sixty-five patients were enrolled and all of them completed the study, N = 33 in NSO and N = 32 in placebo. A significant reduction in Total Nasal Symptom Score and Total Ocular Symptoms Score was observed in the NSO group compared to the placebo, highlighting the potential of NSO in alleviating AR symptoms. The episodes of AR symptoms per day and the frequency of symptoms in 24 hours reduced significantly in 15 days in both groups, but the extent of improvement was significantly higher in NSO compared to placebo. Improvement in Patient Global Impression of Change was also significantly better in NSO compared to the placebo. Serum Immunoglobulin E levels decreased in NSO but were not significantly different from placebo. No clinically significant changes were observed in vital signs, liver and renal function, lipid profile, hematology, fasting blood sugar, or urine analysis at the end of the study.

**Conclusion:**

The result of the study demonstrates that NSO 250 mg with 2.5 mg piperine is an effective and well-tolerated supplement for the management of AR symptoms.

## 1. Introduction

Allergic rhinitis (AR) is a common condition triggered by exposure to environmental aeroallergens, with characteristic symptoms such as sneezing, nasal itching, rhinorrhea (nasal discharge), and obstruction. Despite being perceived as mild, AR imposes substantial individual and societal burdens.^[1]^ AR is estimated to affect around 10% to 30% of the global adult population.^[2,3]^ The prevalence of AR ranges from 3.6% to 54.5% across the different continents.^[4]^

AR is caused by an Immunoglobulin E (IgE)-mediated immune response against inhaled allergens and involves mucosal inflammation driven by the type 2 helper T-cell (Th2) activation. Topical or oral antihistamines, intranasal corticosteroids, intranasal anticholinergics, leukotriene receptor antagonists, and immunotherapy are used for the management of AR.^[5]^ Antihistamines in combination with decongestants, or intranasal corticosteroids are recommended for clinical treatment of AR.^[6]^ Headache, abdominal pain laryngitis, and susceptibility to infections are the side effects associated with leukotriene receptor antagonists and corticosteroids.^[7,8]^ Natural products offer a safer option as an alternate strategy for managing the symptoms and duration of AR.

*Nigella sativa* or black seed has been traditionally used as an herbal medicine, especially in Arabian, African, and Asian systems of medicine. It is well-recognized that black seeds can help manage metabolic diseases, hypertension, allergy, asthma respiratory illness, infectious diseases, celiac diseases, bronchial asthma, headaches, dysentery, infections, obesity, back pain, hypertension, and gastrointestinal issues.^[9–12]^ The oil component of *N sativa*, constituting almost 36% to 38% of the total components contains various pharmacologically active compounds, such as thymoquinone (TQ), thymohydroquinone, dithymoquinone, thymol, carvacrol, nigellidine, nigellicine, and α-hederin, which are responsible for its therapeutic benefits.^[11]^

The pharmacological effect of *N sativa* oil (NSO) and its constituents have been studied in cellular and animal models of respiratory diseases and have been reported to possess antihistaminic, anti-inflammatory, anti-leukotrienes, anti-fibrotic, and immunomodulatory effects.^[13,14]^ *N sativa* seeds were found to be better than montelukast in alleviating the night time symptoms of patients with seasonal AR.^[15]^ Nikakhlagh et al, 2011 reported positive effects of NSO consumption over 4 weeks on the symptoms of AR.^[16]^ Addition of *N sativa* seeds to conventional immunotherapy was reported to increase the CD8-positive cytotoxic cell counts and intracellular killing activities of polymorphonuclear leukocytes in AR patients.^[17]^ TQ, the bioactive compound in *N sativa* seeds was shown to attenuate IgE-mediated allergic response via the Phosphoinositide 3-Kinase–Akt–Nuclear Factor Kappa B pathway by upregulating the nuclear factor erythroid 2-related factor 2–HO-1 axis and decrease the levels of Tumor Necrosis Factor Alpha and Interleukin (IL)-4 in activated Rat Basophilic Leukemia Cells-(RBL- 2H3) cells.^[18]^

Although NSO and its constituent TQ have been shown to have a pharmacological benefit in allergic respiratory diseases, a systematic study using a standardized extract of NSO containing a fixed quantity of TQ is lacking. In the present study, we explored the benefits of NSO standardized for 5% TQ in individuals with seasonal AR.

## 2. Materials and methods

The interventional materials for the study were supplied by Sami-Sabinsa Group Limited. *N sativa* (black cumin) oil was extracted by supercritical fluid extraction and standardized to contain 5% TQ (Nigellin^®^ Amber). The investigational product was a capsule containing NSO (250 mg) with 2.5 mg of 95% piperine (BioPerine^®^). Piperine is a nutritional element recognized for enhancing the absorption of phytochemicals and drugs.^[19,20]^ Microcrystalline cellulose capsule was used as a placebo in this study.

### 2.1. Study design and ethics

This study was conducted as a 2-arm, randomized, double-blind, parallel, and placebo-controlled design at 2 sites, MEDSTAR Speciality Hospital, Sahakarnagar, Bengaluru, and BGS Global Institute of Medical Sciences, Kengeri, Bengaluru, from August 5, 2022, to September 8, 2022. The study was conducted by strictly adhering to the approved ethical guidelines of the Central Drugs Standard Control Organization (September 2019), Indian Council of Medical Research Guidelines 2017, Schedule Y (Drug & Cosmetic Act, India 2014), the World Medical Association Declaration of Helsinki, Fortaleza, 2013 & the International Council for Harmonisation of Technical Requirements for Pharmaceuticals for Human Use (Step V), and “Guidance on Good Clinical Practice” (GCP). The trial was registered prospectively with Clinical Trials Registry - India on 20/07/2022 with the registration number, CTRI/2022/07/044180, and received protocol (CW/106/NIG_ALRH/I/JAN/22) approval from the Institutional Ethics Committee at BGS Global Institute of Medical Sciences and the Medstar Specialty Hospital Ethics Committee. Written voluntary informed consent was obtained from all the participants at the time of enrollment.

### 2.2. Sample size

Based on the earlier study by Kang et al, 2020,^[21]^ the sample size was calculated as 66 participants, at a significance level (alpha) set at 0.05, 80% power, and assuming a correlation of 0.25. Considering a 10% dropout rate, the proposed recruitment target was a total of 72 participants, evenly distributed in a 1:1 ratio between the 2 treatment groups. The details of sample size calculation are provided in the Supplementary Methods section, Supplemental Digital Content, http://links.lww.com/MD/N350.

### 2.3. Study population

#### 2.3.1. Inclusion criteria

The study included individuals aged 18 to 60 years, presenting with at least 2 allergic symptoms such as sneezing, rhinorrhea, nasal obstruction, and nasal itching for a cumulative period exceeding 1 hour per day. These symptoms may be accompanied by tears, itchy, swelling, and red eyes. All the included participants had a documented medical history of AR.

#### 2.3.2. Exclusion criteria

Individuals with known allergic condition like asthma, rhinosinusitis, nasal polyposis or known severe medical conditions, such as cardiovascular, liver or renal dysfunction, diabetes mellitus, cancers, cerebrovascular diseases, and blood system diseases were excluded from the study.

Participants were excluded if they had used steroid, anticoagulant, and immunotherapy within past 1 month. Other exclusion criteria were impaired hematological or liver/renal function, substance abuse, pregnancy or lactation, history of serious allergic reactions to the investigational product, recent clinical trial participation, and any other condition deemed by the principal investigator that could potentially.

Detailed inclusion and exclusion criteria are provided in the Supplementary Methods section, Supplemental Digital Content, http://links.lww.com/MD/N350.

### 2.4. Randomization and blinding

The randomization sequence was prepared by the statistician, using computer-based randomization software (SAS ver 9.4), independent of the sponsoring organization. The principal investigator assigned the participants to 2 groups using the alphanumeric codes. A sealed envelope containing the treatment codes was provided to the principal investigator to be broken only in an emergency, like a serious adverse event. An alpha code was generated for both the active and placebo to improve the blindness of the study and concealment of allocations. Block randomization with a block size of 6 was followed. The participants principal investigator and the sponsors were blinded to the study. The investigational products were packed in a multidose sealed container to ensure the concealing of product identity at all levels.

### 2.5. Intervention

Subjects received either a capsule of NSO 250 mg with BioPerine^®^ 2.5 mg or a placebo capsule twice a day after food for 15 days. Subjects were scheduled for in-person assessments on Day 5 and Day 15 to evaluate study outcome measures. Additionally, telephonic follow-ups were conducted on Day 21 and Day 30 to monitor overall well-being and inquire about any potential adverse events.

### 2.6. Outcome measures and endpoints

The primary endpoints of the study were the mean change in total nasal symptoms score (TNSS) and the mean change in the duration of AR symptoms from baseline to Day 15. Secondary endpoints encompassed measuring the mean change in total ocular symptoms score (TOSS), TNSS, duration of AR symptoms occurring in 24 hours, AR symptom severity, serum levels of IgE, and Patients’ Global Impression of Change (PGIC) scale. This study also evaluated the frequency of the allergic symptoms from baseline to Day 15. The safety parameters were evaluated through monitoring adverse events, and vital signs at each visit, including body temperature, pulse rate, and blood pressure, along with systemic, cardiovascular, respiratory, and central nervous system examinations.

TNSS: It is a brief questionnaire to evaluate the severity of AR symptoms.^[22]^ It consists of questions that assess nasal obstruction, itching/sneezing, and secretion/runny nose, using a 4-point scale from “0” (no symptoms) up to “3” (severe symptoms).

TOSS: This questionnaire was used to measure itchy, redness, tearing (eyes watering), and swelling (puffy eyes).^[23]^ The participant’s response was rated on a 5-point scale ranging from “all of the time” (score 4) to “none of the time” (score 0).

PGIC: The change in activity, limitations, symptoms, emotions, and overall quality of life was recorded by the participants in a 7-point scale ranging from no change scored as 1 and a great deal better (score 7).^[24]^

Serum IgE: The IgE levels were measured by ECLIA, using Cobas e 801 PRO analytical unit (Roche Diagnostics, Basel, Switzerland).

Patient diaries were given to each research participant so they could keep track of their daily allergy symptoms. The frequency and duration of one or more of the symptoms like sneezing, rhinorrhea, nasal blockage and itching, tears, itchy eyes, swelling, and reddening of the eyes were recorded every day. The total frequency was calculated as the number of times any of these symptoms occurred in last 24 hours and the total duration as the time in minutes, for which the symptoms persisted. The severity of the symptoms was recorded as absence, very mild, mild, moderate, severe, and very severe. Percentage was calculated by dividing the number of participants in each class by the total number of participants in the group multiplied by 100.The total frequency was calculated as the number of times any of these symptoms occurred in last 24 hours and the total duration as the time in minutes, for which the symptoms persisted.

Severity of the symptoms were recorded as absence, very mild, mild, moderate, severe, and very severe. Percentage was calculated by dividing the number of participants in each class of severity by the total number of participants in the group multiplied by 100.

### 2.7. Safety measurements

The safety data were monitored throughout the study period by the principal investigator at every visit by physical examination and incidence of unfavorable changes in health. The changes in the pulse rate, body temperature, and clinical biochemical parameters including, the liver profile, renal profile, hematology, and urine analysis levels were monitored from the baseline to the end of the study. Biochemical parameters were analyzed using Cobas C311 analyzer (Roche Diagnostics, Basel, Switzerland), hematological parameters using Pentra XL 80 analyzer (Kyoto, Japan) and urine analysis using Uriplus 200 (Robonic, India).

### 2.8. Statistical analysis

Data from all 65 subjects who completed the study were included in the analysis. For each participant, the Shapiro–Wilk test was performed on raw data at all-time points to ascertain the normality of the distribution. If *P*-value < .05 then the data was considered as not normally distributed. Comparative analysis was performed using the Wilcoxon Signed Rank test for the non-normally distributed data within the group and the results were presented as Median and *P*-value. If the *P*-value was more than .05, the data was considered as normally distributed data, paired *t* test was performed on raw data to compare each visit with the baseline. An unpaired *t* test was performed for the comparative analysis between treatment groups for normally distributed data and the Mann–Whitney *U* test was performed for non-normally distributed data and a *P*-value was presented. Repeated measures ANOVA (RMANOVA) followed by Dunnett multiple comparisons was performed for repeated measure variables to evaluate the within-group change from baseline.

For categorical variables, the frequency and percentage of the population was presented. A descriptive comparison was provided to differentiate the treatment effect between the treatment groups and within treatment groups. All statistical tests were performed using SAS (ver.9.3, NC) at a significance level of α ≤ 0.05.

## 3. Results

### 3.1. Demographic characteristics

**Figure 1.**
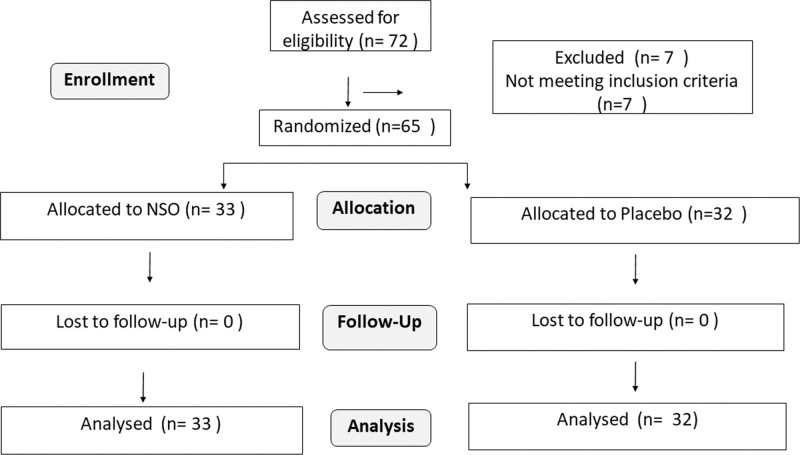
Consort diagram. Flow diagram of the study as per the consolidated standards of reporting trials guideline.

All 65 subjects completed the study, 33 in NSO (250 mg) and 32 in the placebo group (Fig. 1). Participants had a mean age (years) of 33.52 ± 5.56 and all the participants were males. The overall mean values of height, weight, and Body Mass Index were 167.08 ± 6.79 cm, 68.94 ± 11.90 kg, and 24.66 ± 11.90 kg/m^2^ respectively (Table 1).

**Table 1:** Summary statistics of demographics.

Parameter	Active (N = 33)	Placebo (N = 32)	Overall (N = 65)
Age (years)	33.00 ± 4.91	34.06 ± 6.19	33.52 ± 5.56
Range (min, max)	(25.00, 44.00)	(22.00, 44.00)	(22.00, 44.00)
Gender			
Male	33 (100.00 %)	32 (100.00 %)	65 (100.00 %)
Height (cm)	167.24 ± 7.16	166.91 ± 6.49	167.08 ± 6.79
Range (min, max)	(155.00, 185.00)	(153.00, 182.00)	(153.00, 185.00)
Weight (kg)	69.18 ± 12.50	68.68 ± 11.44	68.94 ± 11.90
Range (min, max)	(51.70, 102.90)	(55.90, 108.30)	(51.70, 108.30)
BMI (kg/m^2^)	24.67 ± 3.59	24.66 ± 3.75	24.66 ± 11.90
Range (min, max)	(18.50, 31.40)	(18.80, 34.20)	(18.50, 34.20)

Data are represented as mean ± standard deviation (SD) and range (minimum, maximum) for age, height, weight, and BMI. Gender is represented in number and percentage (%), BMI = body mass index; SD = standard deviation.

### 3.2. Primary efficacy parameters

Table 2 provides a comprehensive overview of the impact of NSO and placebo interventions on TNSS, and TOSS, scores. A significant reduction (*P* < .001) was observed in TNSS from Day 0 (8.45 ± 2.96) to 6.52 ± 2.27 on Day 5 and 4.94 ± 2.25 on Day 15, the in NSO group compared to a minimal change of 7.69 ± 2.63 to 7.28 ± 2.64 and 6.66 ± 2.39 in the placebo group. The mean change was highly significant between NSO and placebo (*P* < .001). Similarly, TOSS also exhibited a substantial decrease in the NSO group from 27.88 ± 15.77 on Day 0 to 21.21 ± 11.26 and 13.07 ± 10.98, on Days 5 and 15 respectively, while the change in placebo was from 32.03 ± 16.48 to 28.13 ± 14.98 and 23.62 ± 13.36 on Days 5 and 15 respectively. The change in TOSS scores was significantly better in NSO compared to placebo (*P* < .001).

**Table 2:** TNSS and TOSS scores at different time points in NSO and placebo.

Parameter	Day 0	Day 5	Day 15	*P*-value*	*P*-value†
TNSS					
Placebo	7.69 ± 2.63	7.28 ± 2.64	6.66 ± 2.39*	.001	<.001
Change from Day 0		0.41	−1.03		
NSO	8.45 ± 2.96	6.52 ± 2.27*	4.94 ± 2.25*	<.001	
Change from Day 0		−1.93	−3.52		
TOSS					
Placebo	32.03 ± 16.48	28.13 ± 14.98	23.62 ± 13.36	<.001	<.001
Change from Day 0		−3.90	−8.41		
NSO	27.88 ± 15.77	21.21 ± 11.26	13.07 ± 10.98	<.001	
Change from Day 0		−6.67	−14.81		

Data are represented as mean ± standard deviation and mean change from Day 0. One-way repeated measure analysis of variance (RMANOVA) was computed for each group at different time points.

TNSS = total nasal symptom score, TOSS = total ocular symptom scale.

* RMANOVA significance within the group.

† Comparison of mean change in value from Day 0 to Day 15 between NSO and placebo.

* *P* < .05 as computed by Post hoc analysis using Dunnett multiple comparisons with Day 0.

### 3.3. AR symptom severity

Table 3 presents summary statistics of AR symptom severity between patients receiving the NSO placebo. At the beginning of the study, all the participants classified their symptoms as either very severe or severe. Over 5 days of supplementation, 27.7% of the participants in NSO and 6.2% in placebo reported a reduction in the severity of the symptoms to a moderate level. At the end of the study in 15 days, 96.7% of the participants in the NSO group felt their symptoms were moderate (48.4%), mild (33.3%) very mild (9.9%), and absence of symptoms (6.1%). In placebo, 34.4% of participants had moderate symptoms and 65.8 % continued to experience severe symptoms (Table 3).

**Table 3:** Summary statistics of AR symptom severity.

Parameter	Active (N = 33)	Placebo (N = 32)	*P*-value
Visit 1 (0 Days)			
Very severe	14 (42.42 %)	7 (21.87 %)	.2396
Severe	19 (57.57 %)	24 (75.00 %)	
Moderate	0 (00.00 %)	1 (3.12 %)	
Visit 2 (5 days)			
Very severe	1 (3.03 %)	3 (9.375 %)	.0561
Severe	23 (69.69 %)	27 (84.37 %)	
Moderate	9 (00.00 %)	2 (6.25 %)	
Visit 3 (15 days)			
Severe	1 (3.03 %)	21 (65.62 %)	.0004
Moderate	16 (48.48 %)	11 (34.37 %)	
Very mild	11 (33.33 %)	0 (00.00 %)	
Mild	3 (9.09 %)	0 (00.00 %)	
Absent	2 (6.06 %)	0 (00.00 %)	

### 3.4. Duration and frequency of AR symptoms

**Figure 2.**
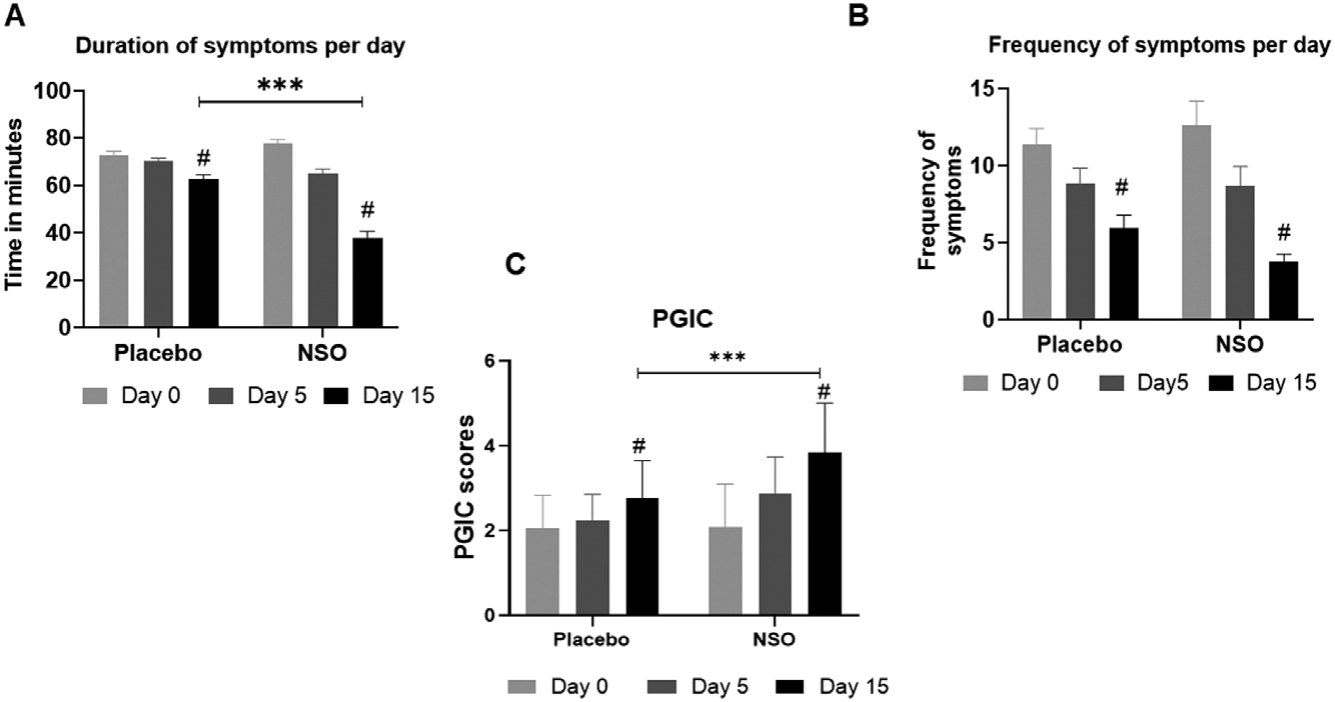
Duration, and frequency of allergic symptoms, and quality of life. The duration of AR symptoms in minutes per day (A), the frequency of the symptoms (B), and the patient’s impression of change in symptoms (C) in the NSO and placebo groups are shown in the figure. ^#^*P* < .01 on Day 15 compared to Day 0, ***P < .001 in NSO compared to placebo.

The mean duration of all the episodes of AR symptoms in 24 hours was 72.97 ± 1.38 minutes in placebo and 77.70 ± 1.74 minutes in NSO at the beginning of the study. This reduced to 65.15 ± 10.19 minutes on Day 5 and 37.73 ± 16.9 minutes on Day 15 in NSO (*P* < .001) and 70.00 ± 8.85 minutes on Day 5 and 62.78 ± 8.87 minutes on Day 15 in placebo. The difference between NSO and placebo was found to be significant (*P* < .001) (Fig. 2A). The frequency of the symptoms per day reduced from 11.81 ± 1.64 to, 8.88 ± 0.96 and 5.88 ± 0.47 in the placebo and from 12.50 ± 1.0 to 8.69 ± 1.25 and 3.53 ± 0.72 in NSO group, the decrease being significant in both groups (Fig. 2B).

### 3.5. Patient’s Global Impression of Change

The participants in the NSO group observed a significant improvement in their condition from the beginning (2.09 ± 1.01) to 2.88 ± 0.86 on Day 5 and 3.85 ± 1.15 at the end of the study. Minor improvements were also observed in placebo (2.06 ± 0.76–2.23 ± 0.65 and 2.77 ± 0.88) on Days 0, 5, and 15 respectively. The difference between NSO and placebo was highly significant (*P* < .001) (Fig. 2C and Table S1, Supplemental Digital Content, http://links.lww.com/MD/N350).

### 3.6. IgE and eosinophils

**Figure 3.**
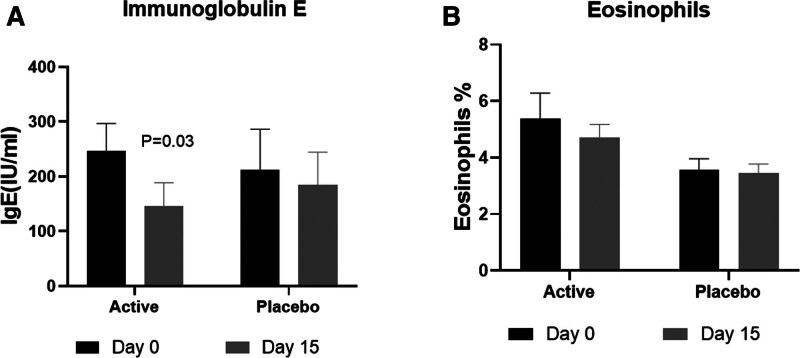
The levels of Immunoglobulin E and eosinophil percentage in peripheral circulation. The serum levels of Immunoglobulin E (IgE) (A) and the eosinophils as percentage of blood cells (B) are presented.

IgE levels demonstrated a noteworthy decrease in the NSO group with a mean change of −100.49 IU/mL, *P* = .03, compared to a slight decrease in the placebo group −26.56 IU/mL (Fig. 3A). The percentage of eosinophils in peripheral blood reduced in both groups, but the difference was not significant (Fig. 3B).

### 3.7. Safety

Clinically significant changes were not observed in the hematological parameters (Tables S2, Supplemental Digital Content, http://links.lww.com/MD/N350), biochemistry (Tables S3, Supplemental Digital Content, http://links.lww.com/MD/N350), urine analysis (Tables S4 Supplemental Digital Content, http://links.lww.com/MD/N350) and vital signs (Tables S5, Supplemental Digital Content, http://links.lww.com/MD/N350) from Day 0 to Day 15 in any participant. All the parameters were in the normal range suggesting the safety of NSO in participants.

## 4. Discussion

The current study reports the clinical effectiveness and safety of *N sativa* oil standardized for 5% TQ in reducing the symptoms and duration of AR in adults with seasonal allergies. Earlier studies have reported that NSO could reduce nasal congestion itching and sneezing attacks in patients with AR.^[16,25]^ The result of this study corroborates with this report and suggests that NSO could be an effective alternative to manage the symptoms of seasonal allergies.

The study revealed noteworthy reductions in nasal symptoms (41.5% vs 13.4%), ocular symptoms (53.1% vs 26.3%), and improvement in the PGIC scale (84.2% vs 34.5%) in the NSO group in comparison to the placebo group at the end of 15 days supplementation. The duration of AR symptoms in 24 hours decreased by 39.2% in the NSO group compared to 24.6% in placebo. An improvement was noted in symptom severity as only 1 subject (3.03%) reported severe AR symptoms at the end of the study compared to 65.62% in the placebo. IgE levels in serum (40.7% vs 12.5%), and the percentage of eosinophils also reduced in the peripheral circulation in these subjects, suggesting an overall improvement in allergic symptoms.

In an earlier study, Nikakhlagh et al, 2011, reported similar effects on the symptoms of AR, by NSO (0.5 mL/day) consumption for 4 weeks. Although the study could not find a statistical difference in serum IgE levels and peripheral eosinophil count, clinical efficacy could be demonstrated.^[16]^ In another study, treatment with NSO (40–80 mg/kg/day for 8 weeks), resulted in a reduction of eosinophil count, IgE, in patients with allergic disorders including rhinitis, atopic eczema, and bronchial asthma.^[25]^

Ansari et al, 2010 demonstrated the comparable efficacy of NS seed extract (250 mg/day) and montelukast in seasonal AR.^[15]^ As an adjuvant to specific immunotherapy NS seeds (2 g/day) were shown to increase the intracellular killing activities of polymorphonuclear leukocytes and also CD8 counts.^[17]^ These studies were carried out with *N sativa* seeds or oil without any standardization for bioactives in contrast to the present study, wherein the NSO was standardized to contain 5% TQ.

AR is a symptomatic reaction induced by exposure to allergens and is mediated by an IgE-mediated hypersensitivity reaction. The allergens activate Th2 lymphocytes which secrete cytokines to activate mast cells, and eosinophils and induce the B-lymphocytes to produce antibodies with IgE isotype.^[26]^ Allergen-stimulated mast cells secrete histamine, prostaglandins, and leukotrienes and induce nasal symptoms. During the late reaction, these cells along with eosinophils, and T cells migrate to the nasal mucosa, destroy the normal nasal tissue, and cause nasal obstruction which is the main symptom of AR patients.^[27,28]^ Treatment of AR is consequently directed towards reducing these mediators.

Animal studies have shown that NSO inhibits the cyclooxygenase and 5-lipoxygenase pathways of arachidonic acid metabolism (Houghton et al, 1995), resulting in the decreased synthesis of thromboxane and leukotrienes. The anti-inflammatory activity of black seed oil in vivo was confirmed in an animal model (Al-Ghamdi, 2001). The attenuation of the inflammatory response of 500 mg/kg NSO was comparable to a 100 mg/kg dose of acetylsalicylic acid (aspirin). The anti-inflammatory and anti-allergic effects of NSO as equally effective as mometasone furoate in the treatment of experimentally generated AR.^[9]^

TQ, the active ingredient in NSO is a potent anti-inflammatory agent and was shown to inhibit allergic airway inflammation by inhibiting Th2 cytokines and eosinophil infiltration into lungs, in a mouse model of allergic lung inflammation.^[29]^ TQ was also shown to have immunomodulatory activities mediated by Nuclear Factor Kappa B and Phosphoinositide 3-Kinase/Akt pathways and antioxidant effect by upregulation of nuclear factor erythroid 2-related factor 2 and HO-1 proteins and decrease the levels of Tumor Necrosis Factor Alpha and IL-4 in activated Rat Basophilic Leukemia Cells-2H3 cells.^[18]^ In a rat peritoneal mast cell in vitro experiment, nigellone, a carbonyl polymer of TQ, demonstrated high effectiveness in inhibiting histamine release.^[30]^ This was in confirmation of an earlier report that suggested that TQ and thymohydroquinone possess significant antihistaminic effects.^[31]^

Thus, the beneficial effects observed in this study are likely to be due to the cumulative effect of the action of NSO and TQ on various mediators of allergic reactions. Further research and clinical investigations are essential to elucidate specific molecular mechanisms and optimize their therapeutic application in AR.

There were no clinically significant changes were observed in vital signs, liver function, renal function, lipid profile, hematology, fasting blood sugar, or urine analysis. These findings strongly support the safety profile of NSO 250 mg, twice a day, indicating its potential as a well-tolerated therapeutic option for AR.

There are a few limitations that need to be highlighted. The study was conducted in a relatively small population in the southern part of India. Further, all the participants in the study were males, although it was not intentional. Earlier studies have shown the efficacy of the oil from *N sativa* seeds on symptoms of allergies in humans. In the present study, we used a standardized extract containing 5% TQ in the oil. Future studies comparing this standardized extract with conventional NSO would also help in understanding the comparative efficacy of the standardized extract. Future studies in a larger and more diverse population of different ethnic backgrounds, and evaluating the inflammatory markers associated with AR would help to understand the benefits and mechanism of action of NSO in seasonal allergies.

## 5. Conclusion

In conclusion, the outcomes of this study suggested a positive impact of NSO standardized for 5% TQ (Nigellin^®^ Amber) in AR caused by seasonal allergens. Further studies in larger populations in different ethnic backgrounds will help to position NSO as an effective and safe supplement to manage the symptoms of AR.

## Acknowledgments

The authors would like to acknowledge the entire clinical research team from the Sami-Sabinsa group, BGS Global Institute of Medical Sciences, Kengeri, Bengaluru, and MEDSTAR Speciality Hospital, Sahakarnagar, Bangalore. The authors thank the statistical team from M/s. Sanjeevani, Bio Services Pvt. Ltd, who independently analyzed the data.

## Author contributions

**Conceptualization:** Anju Majeed.

**Data curation:** Avinash Kadasiddappa Parameswarappa, Lakshmi Mundkur.

**Investigation:** Satish Gudimallam, Chikkalingaiah Siddegowda, Harshith Chandra Shekar.

**Methodology:** Shaheen Majeed, Lakshmi Mundkur.

**Project administration:** Avinash Kadasiddappa Parameswarappa.

**Resources:** Anju Majeed, Shaheen Majeed.

**Supervision:** Avinash Murali, Satish Gudimallam, Lakshmi Mundkur.

**Writing – original draft:** Avinash Murali, Lakshmi Mundkur.

**Writing – review & editing:** Anju Majeed, Shaheen Majeed, Lakshmi Mundkur.

## Supplementary Material
