# Supplementary material for: A randomized, double-blind, placebo-controlled study to evaluate the benefits of a standardized Nigella sativa oil containing 5% thymoquinone in reducing the symptoms of seasonal allergy
Source: Medicine (Baltimore). 2024 Aug 9;103(32):e39243. doi: 10.1097/MD.0000000000039243 (PMC11315530; doi:10.1097/MD.0000000000039243)
Supplement: Supplementary file 1 [file medi-103-e39243-s001.pdf]

Supplementary methods

Sample Size Determination:

The sample size was calculated based on a research publication[1]. As per research publication, baseline values of Total Nasal Symptom Scores

Group 1 baseline-TNSS mean=8.09, SD=1.92

Group 2 baseline-TNSS mean=7.55, SD=1.92

We chose mean=7.82 and SD=1.92 as being towards the center of the two baseline results.

An SD=1.92 with mean=7.82 means the SD is about 0.25 times (base correlation) the size of the mean. That is, the coefficient of variation (CV) is 0.

Using these inputs with 80% power and alpha=0.05 significance level assuming correlation of 0.25, the required total sample size is 66 for evaluation.

Allowing for 10% drop-out rate, the required sample size for recruitment is total of 72 in 1:1 ratio between placebo and NSO, N=36 each

Detailed inclusion and Exclusion criteria

Inclusion criteria

The inclusion criteria were as follows:

- Male or Female in the age of 18 to 60 years.
- Patients with at least 2 or more allergic symptoms: sneezing, rhinorrhoea, nasal obstruction, and nasal itching for a cumulative period greater than 1 hour per day. These symptoms may be accompanied by tears, itchy, swelling & red eyes.
- Patients with a medical history of allergic rhinitis.
- Patients who willing to give written informed consent and comply with requirements of the trial.

Exclusion criteria

The exclusion criteria were as follow:

1. Patients with Known chronic diseases such as asthma, rhinosinusitis, nasal polyposis
2. Patients with Known severe medical conditions, such as cardiovascular, liver, or renal dysfunction, diabetes mellitus, cancers, cerebrovascular diseases, and blood system diseases.
3. Patients with Concomitant steroid, anticoagulant, and immunotherapy within the past 1 month.

4. Patients with Impaired haematological profile and liver / renal function.
5. Patients with known history of alcohol and / or drug abuse.
6. Participants who are pregnant or lactating
7. Patients with a History of serious allergic reaction to investigational product
8. Participant has participated in any clinical trial within the last 3 months
9. Patients with any other condition which the Principal Investigator thinks may jeopardize the study.

Statistical analysis

Statistical analysis was evaluated as per the statistical analysis plan prepared prior to initiation of the study, using SAS (ver.9.3, NC, USA)

Data from all 65 subjects who completed the study were included in the analysis. Shapiro-Wilk test was performed on raw data at all-time points to ascertain the normality of the distribution. If p-value <0.05 then the data was considered as not normally distributed. Comparative analysis was performed using the Wilcoxon Signed Rank test for the non-normally distributed data within the group and the results were presented as Median and p-value. If the p-value was more than 0.05, the data was considered as normally distributed data, paired t-test was performed on raw data to compare each visit with the baseline. An unpaired t-test was performed for the comparative analysis between placebo and NSO groups for normally distributed data and the Mann Whitney U test was performed for non-normally distributed data and a p-value was presented. Repeated measures ANOVA (RMANOVA) followed by Dunnett's multiple comparisons was performed for repeated measure variables to evaluate the within-group change from baseline. For categorical variables, the frequency and percentage of the population was presented. A descriptive comparison was provided to differentiate the treatment effect between the treatment groups and within treatment groups. All statistical tests have used a significance level of $\alpha \leq 0.05$.

Laboratory data are summarized by presenting summary statistics of raw data and change from screening values (means, medians, SDs, SEM) as well as shift tables showing change from screening to end of study in laboratory values relative to normal reference limits.

APPENDIX – II: TOTAL NASAL SYMPTOM SCORE

PLEASE ANSWER ALL QUESTIONS TO THE BEST OF YOUR ABILITY. This information will assist us inunderstanding and treating your symptoms.

1. Please rate how your **nasal congestion** has been over the past:

	12 hours	Last 2 weeks
None	0 ○	0 ○
Mild (symptom clearly present but easily tolerated)	1 ○	1 ○
Moderate (symptom bothersome but tolerable)	2 ○	2 ○
Severe (symptom difficult to tolerate – interferes with activities)	3 ○	3 ○

2. Please rate how your **runny nose** has been over the past:

	12 hours	Last 2 weeks
None	0 ○	0 ○
Mild (symptom clearly present but easily tolerated)	1 ○	1 ○
Moderate (symptom bothersome but tolerable)	2 ○	2 ○
Severe (symptom difficult to tolerate – interferes with activities)	3 ○	3 ○

3. Please rate how your **nasal itching** has been over the past:

	12 hours	Last 2 weeks
None	0 ○	0 ○
Mild (symptom clearly present but easily tolerated)	1 ○	1 ○
Moderate (symptom bothersome but tolerable)	2 ○	2 ○
Severe (symptom difficult to tolerate – interferes with activities)	3 ○	3 ○

4. Please rate how your **sneezing** has been over the past:

	12 hours	Last 2 weeks
None	0 ○	0 ○
Mild (symptom clearly present but easily tolerated)	1 ○	1 ○
Moderate (symptom bothersome but tolerable)	2 ○	2 ○
Severe (symptom difficult to tolerate – interferes with activities)	3 ○	3 ○

5. Please rate how **difficult sleep** has been with nasal symptoms:

	Last night	Last 2 weeks
None	0 ○	0 ○
Mild (symptom clearly present but easily tolerated)	1 ○	1 ○
Moderate (symptom bothersome but tolerable)	2 ○	2 ○
Severe (symptom difficult to tolerate – interferes with activities)	3 ○	3 ○

Reference

1. Kang M-G, Han S-W, Kang H-R, Hong S-J, Kim D-H, Choi J-H. Probiotic NVP-1703 alleviates allergic rhinitis by inducing IL-10 expression: a four-week clinical trial. *Nutrients*. 2020;12(5):1427.

Table S1: PGIC and IgE scores at different time points in NSO and Placebo

**Parameter**	**Day 0**	**Day 15**	**P-value[a]**	**P-value[b]**
		**PGIC**		
	2.06±0.76	2.77±0.88	0.001	0.0005
		0.63		
	2.09±1.01	3.85±1.15	<0.001	
		1.76		
		**IgE IU/mL**		
Placebo	211.85±74.77	185.28±59.2	0.07	0.051
Change from day 0		-26.56		
NSO	247.03±49.75	146.54±42.1	0.03	
Change from day 0		-100.49		

Data are represented as Mean ± Standard deviation for PGIC and Mean and Standard error of mean for IgE levels. P-value[a]: significance within the groups from Day 0 to Day15 by Wilcoxon test, P-value[b] : Significance between the group by Mann Whitney test. PGIC: Patients Global Impression of Change Questionnaire

Table S2: Summary Statistics of Hematological parameters

Parameters (Mean±SD)	Group	Visit 1	Visit 3	Inter Group p-value	
				Visit 1	Visit 3
Hemoglobin	*Nigella sativa* oil	14.07±1.87	14.26±1.91	0.9828	0.1610
	Placebo	14.06±1.86	13.61±1.78		
RBC	*Nigella sativa* oil	5.28±0.51	5.12±0.48	0.9520	0.3934
	Placebo	5.27±0.53	5.24±0.57		
Total Leukocyte count	*Nigella sativa* oil	6738.53±1377.12	6705.00±1768.95	0.1647	0.3788
	Placebo	6267.16±1842.51	6288.75±2012.60		
Lymphocytes	*Nigella sativa* oil	31.81±6.21	33.29±8.94	0.6032	0.9735
	Placebo	30.87±8.19	33.36±7.93		
Monocytes	*Nigella sativa* oil	6.49±2.11	7.41±3.28	0.7869	0.3911
	Placebo	6.66±2.89	6.75±2.86		
Neutrophils	*Nigella sativa* oil	56.55±8.21	54.37±8.97	0.3322	0.4670
	Placebo	58.70±9.50	56.15±9.53		
Eosinophils	*Nigella sativa* oil	5.37±4.97	4.71±2.65	0.0951	0.0675
	Placebo	3.57±2.15	3.44±1.89		
Basophils	*Nigella sativa* oil	0.24±0.25	0.22±0.20	0.7658	0.2502
	Placebo	0.19±0.12	0.28±0.23		
MCH	*Nigella sativa* oil	26.83±2.98	27.53±3.57	0.9943	0.1514
	Placebo	26.88±3.68	26.27±3.42		
MCHC	*Nigella sativa* oil	30.80±1.48	31.21±1.70	0.5139	0.1632
	Placebo	30.24±2.17	30.61±1.51		
MCV	*Nigella sativa* oil	86.92±6.96	97.95±8.37	0.7960	<0.0001
	Placebo	87.81±9.31	85.58±8.03		
Platelet Count	*Nigella sativa* oil	3.01±1.03	2.96±1.02	0.9313	0.8089
	Placebo	2.89±0.90	2.90±0.97		
Mean Platelet Volume	*Nigella sativa* oil	10.87±0.84	10.48±0.70	0.6079	0.9640
	Placebo	10.76±0.88	10.47±1.05		

Packed Cell Volume	*Nigella sativa* oil	45.57±4.67	45.71±5.49	0.7425	0.2683
	Placebo	45.95±4.61	44.34±4.31		
HDL_C	*Nigella sativa* oil	42.77±27.73	38.36±12.90	0.3112	0.1912
	Placebo	41.50±10.43	39.56±8.12		
LDL_C	*Nigella sativa* oil	122.70±75.28	110.36±33.61	0.5863	0.2974
	Placebo	118.80±35.60	119.47±36.28		
VLDL_C	*Nigella sativa* oil	28.54±11.06	26.20±9.48	0.6807	0.8759
	Placebo	27.48±9.73	26.56±9.02		
Total Cholesterol	*Nigella sativa* oil	181.27±34.99	178.55±27.42	0.6823	0.9271
	Placebo	184.87±35.59	181.13±37.58		
Triglycerides	*Nigella sativa* oil	168.50±77.40	180.12±106.66	0.9383	0.3192
	Placebo	167.07±70.73	196.69±92.91		

The values related to the systolic blood pressure, and diastolic blood pressure, body temperature and pulse rate from Visit 1 to the end of the study are represented as mean ± SD. SD: standard deviation. MCH: Mean corpuscular hemoglobin, MCHC: mean corpuscular hemoglobin concentration, MCV: mean corpuscular volume, RBC: Red blood cell count, HDL_C: High-density lipoprotein cholesterol, LDL_C: Low-density lipoprotein cholesterol, VLDL_C: Low-density lipoprotein cholesterol. All the haematological parameters were within the normal limits.

Table S3: Summary Statistics of Biochemical parameters

Parameters (Mean±SD)	Group	Visit 1	Visit 3	Inter Group p-value	
				Visit 1	Visit 3
SGOT	*Nigella sativa* oil	22.10±9.68	22.30±15.10	0.6058	0.0344
	Placebo	23.87±15.94	29.66±24.37		
SGPT	*Nigella sativa* oil	24.63±15.85	22.42±17.82	0.8134	0.0948
	Placebo	25.13±20.14	31.97±30.36		
Total Bilirubin	*Nigella sativa* oil	0.64±0.50	0.60± 0.44	0.4501	0.6259
	Placebo	0.75±0.56	0.63±0.50		
FBS	*Nigella sativa* oil	88.33±26.03	87.39±49.98	0.4712	0.3067
	Placebo	86.07±29.45	87.19±27.06		
Alkaline Phosphatase	*Nigella sativa* oil	90.97±22.07	88.36±20.81	0.6857	0.5524
	Placebo	87.57±17.09	89.25±15.38		
Serum Creatinine	*Nigella sativa* oil	0.83±0.11	0.86±0.10	0.0717	0.6063
	Placebo	0.88±0.11	0.89±0.10		
Chloride	*Nigella sativa* oil	103.53±2.49	102.45±2.29	0.3889	0.2637
	Placebo	104.13±2.00	103.16±2.77		
Sodium	*Nigella sativa* oil	139.00±1.68	139.42±1.44	0.0672	0.0330
	Placebo	139.90±1.77	140.28±1.90		
Potassium	*Nigella sativa* oil	4.65±0.67	4.33±0.43	0.7849	0.1657
	Placebo	4.65±0.58	5.71±7.00		
Specific Gravity	*Nigella sativa* oil	1.02±0.01	1.02±0.01	0.0910	0.2484
	Placebo	1.02±0.01	1.02±0.01		

Data represented as Mean±SD. SGOT: Serum Glutamic Oxaloacetic Transaminase, SGPT: Serum Glutamic Pyruvic Transaminase, FBS: Fasting Blood Sugar, SD: Standard deviation. All the biochemical parameters were within the normal limits.

Table S4: Summary Statistics of Urine analysis

Parameters (Mean±SD)	Group	Visit 1	Visit 3	Inter Group p-value	
				Visit 1	Visit 3
eGFR	*Nigella sativa* oil	73.05±29.66	75.97±30.70	0.7214	0.6592
	Placebo	69.56±18.33	70.70±21.41		
Uric Acid	*Nigella sativa* oil	5.19±0.72	5.23± 1.15	0.3252	0.2003
	Placebo	5.40±0.98	6.69±7.04		
Urea	*Nigella sativa* oil	18.50±5.75	18.52±6.25	0.7051	0.9112
	Placebo	19.00±5.90	17.75±5.17		
PH	*Nigella sativa* oil	5.84±0.62	5.72±0.59	0.7508	0.1454
	Placebo	5.72±0.36	5.55±0.55		

Data represented as Mean±SD. eGFR: estimated glomerular filtration rate. All the parameters from urine analysis were within the normal limits.

Table S5: Summary Statistics of Vital parameters

Parameters (Mean±SD)	Group	Visit 1	Visit 3	Inter Group p-value	
				Visit 1	Visit 3
Systolic Blood Pressure (mmHg)	*Nigella sativa* oil	120.06±6.83	120.45±6.96	0.4023	0.8698
	Placebo	121.13±7.24	120.06±7.22		
Diastolic Blood Pressure (mmHg)	*Nigella sativa* oil	76.27±6.93	76.45±6.14	0.9738	0.3021
	Placebo	76.13±7.13	78.03±5.89		
Body Temperature (°F)	*Nigella sativa* oil	97.60±0.62	97.77±0.57	0.7375	0.5387
	Placebo	97.61±0.69	97.63±0.76		
Pulse Rate (beats/min)	*Nigella sativa* oil	77.42±7.11	76.85±6.69	0.5518	0.0131
	Placebo	78.91±8.43	82.63±7.60		

The values related to the systolic blood pressure, and diastolic blood pressure, body temperature and pulse rate from Visit 1 to the end of the study are represented as Mean ± SD. SD: Standard deviation. All the vital parameters were within the normal limits.
